# Evaluation of ATM protein expression in canine mammary tumors

**DOI:** 10.1186/1753-6561-7-S2-P68

**Published:** 2013-04-04

**Authors:** Talita MM Raposo, Carlos E Fonseca-Alves, Erika M Terra, Renata C Bueno, Silvia R Rogatto, Renée Laufer-Amorim

**Affiliations:** 1 Department of Veterinary Clinic and Surgery, UNESP, Jaboticabal, SP, Brazil; 2 Department of Veterinary Clinic, UNESP, Botucatu, SP, Brazil; 3 Institute of Biosciences, UNESP, Botucatu, SP, Brazil; 4 Department of Urology, Faculty of Medicine, UNESP, Botucatu, SP, Brazil

## Background

Ataxia telangiectasia mutated (*ATM*) synthesizes a protein kinase known as a major regulator of DNA damage response. ATM mutations in women have been associated with moderate risk to develop familial Breast Cancer. ATM transcript and protein down-regulation have been reported in sporadic breast carcinomas and the absence of ATM protein expression was also significantly associated with distant metastasis in women. Canine mammary tumors have an incidence three times higher than women and their biological behavior is similar in both species. The aim of this study was to identify the ATM protein expression in canine breast and compared the results with what occurs in women.

## Patients and methods

In this study, we evaluated ATM protein expression by immunohistochemistry of 48 canine breasts samples, and compared ATM expression among normal breasts, benign mammary tumors (hyperplasia or adenoma), non-metastatic and metastatic mammary carcinomas. Evaluation of ATM protein expression was performed by the distribution of the positive cells (score 1: <25% cells positive, 2: 26% to 50%, 3: 51% to 75% and 4:> 75%).

## Results

Kruskal-Wallis test and Wilcoxon test were used (*P* < 0.05). Lower ATM levels were significantly associated with non-metastatic and metastatic mammary carcinoma when compared to normal breast tissue and benign mammary tumors.

## Conclusions

A similar ATM expression was found between non-metastatic and metastatic mammary carcinoma samples and this fact can be explained by the possibility that these patients could present distant metastasis in the future, once they have being monitored for just one year. These data suggests that ATM have a similar behavior in bitches and women.

## Financial support

FAPESP.

